# Correction: Accumulation of Oxidized LDL in the Tendon Tissues of C57BL/6 or Apolipoprotein E Knock-Out Mice That Consume a High Fat Diet: Potential Impact on Tendon Health

**DOI:** 10.1371/journal.pone.0123280

**Published:** 2015-04-02

**Authors:** The PLOS ONE Staff

## Notice of Republication

This article was republished on February 25, 2015, to correct an error in the byline of the online version and the affiliations in the online and PDF versions. The publisher apologizes for the errors. Please download this article again to view the correct version. The originally published, uncorrected article and the republished, corrected article are provided here for reference.

## Supporting Information

S1 File Originally published, uncorrected article. (PDF)

S2 File Republished, corrected article. (PDF)
