# Supplementary material for: Correction: Accumulation of Oxidized LDL in the Tendon Tissues of C57BL/6 or Apolipoprotein E Knock-Out Mice That Consume a High Fat Diet: Potential Impact on Tendon Health
Source: PLoS One. 2015 Apr 2;10(4):e0123280. doi: 10.1371/journal.pone.0123280 (PMC4383611; doi:10.1371/journal.pone.0123280)
Supplement: S2 File — (PDF) [file pone.0123280.s002.pdf]

PLOS ONE

RESEARCH ARTICLE

Accumulation of Oxidized LDL in the Tendon Tissues of C57BL/6 or Apolipoprotein E Knock-Out Mice That Consume a High Fat Diet: Potential Impact on Tendon Health

Navdeep Grewal[1,2], Gail M. Thornton[3,4], Hayedeh Behzad[1,2], Aishwariya Sharma[1,2], Alex Lu[2], Peng Zhang[5], W. Darlene Reid[6], David J. Granville[7,8], Alex Scott[1,2]*

1. Department of Physical Therapy, Faculty of Medicine, University of British Columbia, Vancouver, BC, Canada, **2.** Centre for Hip Health and Mobility, Vancouver Coastal Health Research Institute, Vancouver, BC, Canada, **3.** McCaig Institute for Bone and Joint Health, University of Calgary, Calgary, AB, Canada, **4.** Department of Orthopaedics, Faculty of Medicine, University of British Columbia, Vancouver, BC, Canada, **5.** Deptartment of Medicine, University of British Columbia and Vancouver Coastal Health Research Institute, Jack Bell Research Centre, Vancouver, BC, Canada, **6.** Department of Physical Therapy, University of Toronto, Toronto, ON, Canada, **7.** Department of Pathology and Laboratory Medicine, University of British Columbia, Vancouver, BC, Canada, **8.** Institute for Heart + Lung Health, St. Paul's Hospital, Vancouver, BC, Canada

*ascott@mail.ubc.ca

CrossMark click for updates

OPEN ACCESS

Citation: Grewal N, Thornton GM, Behzad H, Sharma A, Lu A, et al. (2014) Accumulation of Oxidized LDL in the Tendon Tissues of C57BL/6 or Apolipoprotein E Knock-Out Mice That Consume a High Fat Diet: Potential Impact on Tendon Health. PLoS ONE 9(12): e114214. doi:10.1371/journal.pone.0114214

Editor: Hazel RC. Screen, Queen Mary University of London, United Kingdom

Received: May 9, 2014

Accepted: November 3, 2014

Published: December 12, 2014

Copyright: © 2014 Grewal et al. This is an open-access article distributed under the terms of the Creative Commons Attribution License, which permits unrestricted use, distribution, and reproduction in any medium, provided the original author and source are credited.

Data Availability: The authors confirm that all data underlying the findings are fully available without restriction. All relevant data are within the paper and its Supporting Information files.

Funding: A. Scott received grant funding from the Canadian Institutes of Health Research (www.cihr.ca), Institute of Musculoskeletal Health and Arthritis Priority Announcement (126604-1) and a Michael Smith Clinical Scholar Award. The funders had no role in study design, data collection and analysis, decision to publish, or preparation of the manuscript.

Competing Interests: The authors have declared that no competing interests exist.

Abstract

Objective: Clinical studies have suggested an association between dyslipidemia and tendon injuries or chronic tendon pain; the mechanisms underlying this association are not yet known. The objectives of this study were (1) to evaluate the impact of a high fat diet on the function of load-bearing tendons and on the distribution in tendons of oxidized low density lipoprotein (oxLDL), and (2) to examine the effect of oxLDL on tendon fibroblast proliferation and gene expression.
Methods: Gene expression (*Mmp2, Tgfb1*, *Col1a1*, *Col3a1*), fat content (Oil Red O staining), oxLDL levels (immunohistochemistry) and tendon biomechanical properties were examined in mice (C57Bl/6 or ApoE -/-) receiving a standard or a high fat diet. Human tendon fibroblast proliferation and gene expression (*COL1A1*, *COL3A1*, *MMP2*) were examined following oxLDL exposure.
Results: In both types of mice (C57Bl/6 or ApoE -/-), consumption of a high fat diet led to a marked increase in oxLDL deposition in the load-bearing extracellular matrix of the tendon. The consumption of a high fat diet also reduced the failure stress and load of the patellar tendon in both mouse types, and increased *Mmp2* expression. ApoE -/- mice exhibited more pronounced reductions in tendon function

than wild-type mice, and decreased expression of *Col1a1* compared to wild type mice. Human tendon fibroblasts responded to oxLDL by increasing their proliferation and their mRNA levels of *MMP2*, while decreasing their mRNA levels for *COL1A1* and *COL3A1*.

Conclusion: The consumption of a high fat diet resulted in deleterious changes in tendon function, and these changes may be explained in part by the effects of oxLDL, which induced a proliferative, matrix-degrading phenotype in human tenocytes.

Introduction

Chronic tendinopathy is a widespread, age-related condition. Much attention has been given to sport or occupation as risk factors, but there remains an unexplained association of tendinopathy and adiposity, both in active and sedentary populations [1]. Adiposity is also associated with hyperlipidemia, a spectrum of plasma lipid abnormalities including elevated total cholesterol (TC), and elevated levels of low density lipoprotein (LDL-C). Lipid metabolism is regulated by a variety of physiological and pathophysiological pathways with as many as 95 genetic loci linked to lipid pathophysiology [2]. In addition to the consumption of a "Western style" diet rich in saturated fats, defects in genes involved in the synthesis or processing of lipoproteins such as LDL (the most cholesterol-enriched lipoprotein) can cause cholesterol to accumulate in the cells and extracellular matrix of the vasculature, leading to cardiovascular disease. A previous study has shown that average TC and LDL-C were significantly higher in individuals who sustained an Achilles tendon injury compared to a control group, while high-density lipoprotein cholesterol concentration was significantly lower [3]. Abboud and Kim reported similarly that rotator cuff tendinopathy patients demonstrate increased TC and LDL-C compared to controls [4]. Surprisingly, mechanisms linking high fat diet and tendon pathology have not been directly examined.

ApoE deficiency is a commonly used laboratory model of hypercholesterolemia. ApoE is required for normal catabolism and clearance of lipoprotein constituents, acting as a ligand for cell-surface LDL receptors; ApoE -/- mice therefore experience a severe, progressive form of hypercholesterolemia, making them a common choice when studying the adverse influence of cholesterol on various body tissues. ApoE -/- mice (like individuals with ApoE linked familial hypercholesterolemia) develop xanthomas — connective tissue deposits of lipid containing high levels of cholesterol and LDL and increased numbers of macrophages [5, 6]. The LDL component of xanthomas binds primarily to sulphated glycosaminoglycan [5] (which are highly enriched in tendon, being intimately associated with the collagen-rich matrix [7]. The subsequent oxidation of LDL can induce a variety of signaling events in exposed cells, including

upregulated expression of MMP 2 [8] and type I collagen [9], both of which are known to occur in tendinopathic tendon [10–13]. Despite the above, the tendons of ApoE mice have not been extensively studied, and neither has the potential role of oxLDL on tendon health.

Our objectives were (1) to examine the impact of a high fat diet in mice on tendon oxLDL accumulation and tendon health (biomechanical function), and (2) to assess the effect of oxLDL on human tendon fibroblast function (proliferation and gene expression).

Methods

All research involving human participants was approved by the authors' Institutional Review Board (IRB), and all clinical investigation was conducted according to the principles expressed in the Declaration of Helsinki. Informed written consent was obtained from the participants. The UBC Clinical Research Ethics Board reviewed and approved the human studies, and the UBC Animal Care Committee reviewed and approved the mouse studies.

Mice

Animal breeding and experimental procedures were approved by the local Animal Care Committee at the University of British Columbia (protocol #A11-0026 and A11-0027), and were carried out in accordance with the principles and standards of the Canadian Council on Animal Care. Controls (C57Bl/6 mice) were purchased from Charles River Laboratories (Wilmington, MA, USA) and ApoE -/- breeder mice were purchased from the Jackson Laboratory (Bar Harbor, ME, USA). Mouse husbandry was carried out by certified animal laboratory technicians. Mice were maintained on a 24 hr light/dark cycle at room temperature in group housing (4 per cage). Cages contained nesting material and environmental enrichment (plastic tubes, chew toys). In the order that the breeding program yielded the mice, when they reached 7 weeks of age they were randomly assigned to be fed either a high fat "Western diet" (21.2% fat, with >60% of fatty acids being saturated, TD.88137, Harlan Tekland, Madison, WI, USA) or regular chow (5% fat, PicoLab Mouse Diet 20: 5053, LabDiet; Richmond, IN, USA) for a further 15 or 30 weeks, ensuring an equal balance of males and females in each experimental group. Weights were measured on a subset of animals used for this study. The average starting weights of the mice were 25.9 g (ApoE -/-, range 20.1–29, n=31) and 23.3 g (C57Bl/6, range 21.0–27.6 g, n=25). At the time of randomization to receive high or low fat diet (i.e., baseline), the average weights (mean, SD) of the animals were 26 (2.3) for ApoE -/- normal diet (n=13), 25 (1.7) for ApoE -/- high fat diet (n=18); and 22 (0.8) for C57Bl/6 normal diet (n=12) and 24 (1.8) C57Bl/6 high fat diet (n=13).

Mice were euthanized using carbon dioxide, following standard operating procedures for either adult or neonatal rodents, approved by University of British

Columbia. Two tail tendons, two patellar tendons, and two Achilles tendon were harvested from each mouse. For each assay, the individual mouse was defined as the experimental unit. We did not conduct a power analysis, but based on previous experience with histology and biomechanical analysis we desired at least 60 animals (10 animals of each strain at each of three time points).

Histology

Sixty five mice were used (ApoE -/-: n=30; C57Bl/6: n=35). Data were obtained and analyzed from one tendon (either left or right) demonstrating the least amount of artefact (i.e.minimal folding and tearing) from each animal, for all animals except for one ApoE animal (final n=29) due to harvesting and sectioning artefacts. Achilles tendons were removed after sacrificing at three time points (ApoE -/- vs C57Bl/6: baseline, n=11/11; 15 weeks, n=9/9; 30 weeks, n=10/15) with the gastrocnemius muscles attached, frozen with Tissue Tek cryoprotectant embedding medium, and stored at -20℃ until further processing. H&E and Oil Red O slides were prepared using standard protocols, and all investigators were blinded to the identity of the slides by masking the slides with thick tape, and assigning each slide a code using a random number generator. Digital scans were created with Aperio Scancope XT (Aperio Technologies, Vista, CA, USA). Oil Red O staining was quantitated as the positivity ratio (area of positive (red) pixels divided by the total tissue area (all pixels).

Immunohistochemistry

After conducting the histological analysis, twenty-one of the above mice at the 30 week time point had tissue remaining which could be used for immunohistochemistry (10 ApoE -/-: 5 normal and 5 high fat diet: and 11 C57Bl6: 4 normal and 7 high fat). Frozen mouse Achilles tendon tissue sections were fixed in precooled acetone (−20℃) and endogenous peroxidase activity was blocked in 0.3% H_2O_2. Non-specific binding was then blocked with 1% bovine serum albumin and 0.1% Tween 20 in phosphate buffered saline (PBS). A primary antibody against oxLDL (Avanti 330001S, 1:100) was applied, followed by a biotinylated secondary antibody (Abcam ab5929, 1:500) and an Avidin/Biotinylated enzyme Complex (ABC Reagent, Vectastain PK-4002) using 3,3'-diaminobenzidine (DAB, Dako K1497) as the substrate. Mouse atherosclerotic vessels, and isotype controls (IgM, Abcam ab18401) were included in each experiment. Sections were imaged with a Zeiss Axiophot upright light microscope (Carl Zeiss Microscopy, Thornwood, NY, USA) by an investigator who blinded to the animals' dietary groupings. A color deconvolution step calibrated to the DAB chromogen was used. Subsequent images were thresholded with an Otsu method to remove background hue [14], and the area of DAB dye measured. The ratio of DAB to tissue area was calculated, similar to the procedure described for Oil Red O.

Cross-sectional Area (CSA)

From the same 65 mice enumerated above (histology section), the patellar tendons and their bony insertions were dissected from both limbs, wrapped in PBS-soaked gauze and frozen at -20℃ until testing. The patella and tibia were clamped and the specimen submersed in PBS. The tendon was scanned in a transverse direction using a CLI 1500 Water-Path Diagnostic Ultrasound Probe 35 MHz transducer (E-Technologies, Inc., Davenport, IA, USA). Images captured using Reflex Ultrasound Bio-Microscope software (E-Technologies, Inc., Davenport, IA, USA) were digitally traced to determine CSA. Data from all animals was included in the analysis. The standard deviation of the difference between replicate CSA measures was 0.118 mm^2 (n=127 paired measurements).

We used a near-field ultrasound phantom (CIRS 050) to calibrate the US unit and probe used in this study, and to estimate the resolution of the obtained digital images. The nearest two horizontally placed objects (0.1 mm steel pins) in the CIRS 050 phantom were 0.2 mm, and these two pins could be clearly resolved. Therefore, we calculated the theoretical resolution by calculating the FWHM (full width half maximum) of the pixel brightness in the vertical and horizontal dimensions. Vertical resolution was 0.159 mm and horizontal was 0.191 mm.

RNA Isolation and Quantitative PCR

Tail tendons from the animals described above were stored in RNA preservative (RNAlater) at -20℃. A subset of tendons from animals at the 30 week time point (10 ApoE -/- (7 normal diet, 3 high fat), 15 C57Bl/6 (7 normal diet, 8 high fat)) was randomly selected and total RNA was extracted and purified as described previously [15]. qPCR was conducted in technical triplicates, with SYBR green primers sets for relevant genes (Table 1) was conducted (ABI 7500 Fast system) and values normalized to *Gapdh*, which did not significantly vary across experimental conditions. Relative quantities of mRNA for *Mmp2, Col1a1, Col3a1* and *Tgfb* were computed using the $2^{-\Delta Ct}$ method.

Biomechanical testing

The right and left patellar tendons of ApoE -/- and C57Bl/6 (n=65, detailed above) were tested to failure using Bose ElectroForce 5100 Biodynamic test instrument (Bose Corporation, Eden Prairie, MN, USA). This resulted in 130 biomechanical tests, of which 120 were successfully conducted; the remaining 10 tests (7 C57Bl/6, 3 ApoE -/-) were terminated unsuccessfully due to damage to the specimen during dissection or mounting. The tibia of the tibia-patellar tendon-patella complex was moulded in Wood's metal in an aluminum block. The patella was held with custom-made grips. In load control mode, each tendon was preloaded to 0.5 N and the initial length of the tendon measured with digital calipers prior to preconditioning with loads of 0.5 N to 1.5 N at a frequency of 2 Hz for 10 cycles. In displacement control mode, a constant ramp-to-failure test was performed at a rate of 0.167 mm/s. Strain was calculated by dividing the

Table 1. Primer sequences for qPCR.

Gene*	Forward Primer	Reverse Primer
Tgfb	GCTGAACCAAGGAGACGGAA	ATGTCATGGATGGTGCCCAG
Col1a1	CGATGGATTCCCGTTCGAGT	GAGGCCTCGGTGGACATTAG
Col3a1	CAGGAGAAAAGGGTCCTCCC	ATACCCCGTATCCCTGGACC
Gapdh	AAGGGCTCATGACCACAGTC	CAGGGATGATGTTCTGGGCA
COL1A1	TGTTCAGCTTTGTGGACCTCCG	CGCAGGTGATTGGTGGGATGTCT
COL3A1	AATCAGGTAGACCCGGACGA	TTCGTCCATCGAAGCCTCTG
MMP2	GAGTGCATGAACCAACCAGC	GTGTTCAGGTATTGCATGTGCT
GAPDH	TCTTTTGCGTCGCCAGCCGAG	TGACCAGGCGCCCAATACGAC

*Mouse gene symbols are in lower case, human genes in upper case.

doi:10.1371/journal.pone.0114214.t001

displacement by the initial length of tendon, while stress was calculated by dividing the load by initial cross-sectional area. Failure load and failure stress were defined as the peak values of the load-displacement and stress-strain curves, respectively. The failure strain was defined as the maximum strain recorded during the failure test. Tangent stiffness was the slope of the linear regression over the range of 35%–60% of the failure load of the load-displacement curve. Modulus was calculated using the same range. Failure load, failure stress, failure strain, stiffness, and modulus were calculated for each group. The failure loads reported in the current study for patellar tendons from C57Bl/6 mice were within the range of 9.15 N +/−1.93 N reported by Sereysky et al. [16].

Preparation of oxLDL

Low density lipoprotein (LDL, d=1.019–1.063 g/mL) was isolated by sequential ultracentrifugation from EDTA-anticoagulated whole blood collected from 2 healthy human normolipidemic donors after 12 hours of fasting [17]. LDL was then dialyzed to reduce EDTA concentration to 10 µM. Oxidation was achieved by incubating 200 µg/mL LDL in DPBS containing 0.90 mmol/L CaCl2 and 0.49 mmol/L MgCl2 with 5 µM copper sulfate at 37℃ for 24 hours. The reaction was stopped by addition of 40 µM butylated hydroxytoluene (BHT) and 300 µM EDTA. The oxLDL was then washed and concentrated to approximately 1.5 mg/ml using Amicon Ultra-15 filter unit with Ultracel PL-30 membrane (Millipore, Billerica, MA, USA). The oxLDL was filtered through 0.45-micron filters and the protein concentrations were measured using the bicinchoninic acid protein assay. The oxLDL from the two donors was not pooled, but was used separately as biological duplicates to confirm the observed effects.

Human tenocyte culture experiments

Human tendon cells were derived and grown exactly as described previously [18]. Tenocytes were grown in Dulbecco's modified Eagle's medium (DMEM) containing 2% fetal bovine serum (FBS) overnight. Cells were treated with oxLDL

at 10 μg/ml, 15 μg/ml, 25 μg/ml, and 50 μg/ml in DMEM plus 2% FBS for 48 hrs. For control, cells were treated with the same media that was used to dissolve oxLDL. Tenocyte proliferation was assessed with the 3-(4,5-dimethylthiazol-2-yl)-5-(3-carboxymethoxyphenyl)-2-(4-sulfophenyl)-2H-tetrazolium salt (MTS) assay. The absorbance at 490 nm was recorded with a spectrophotometer and the absorbance of oxLDL treated cells expressed as the percentage proliferation relative to control. qPCR for *MMP2*, *COL1A1* and *COL3A1* was carried out as described above with the forward and reverse primers (human) listed in Table 1. Experiments were carried out in triplicates and each entire experiment was independently replicated.

Statistical analysis

A linear model was used to analyze differences in weight among ApoE -/- and C57Bl/6, normal and high fat diets, at the 15 week and 30 week time points. For cross-sectional area and biomechanical variables, Oil Red O staining, and mRNA levels, a linear mixed model was used to determine if there were any significant differences according to mouse type (ApoE -/- vs. C57Bl/6) or diet (regular chow vs. high fat) at 15 and 30 weeks. Normal quantile plots and residual plots were graphed to ensure the assumptions of the tests were met. ANOVA was used to examine differences in human tenocyte proliferation or mRNA levels exposed to different doses of oxLDL. Means and standard deviations are shown unless otherwise indicated. Box plots show a center bar as the median, boxes as the upper and lower quartiles, and whiskers to indicate the minimum and maximum.

Results

Mouse weight

Both strains of mice (ApoE -/- and wild type) were significantly heavier at the conclusion of the experiment (30 week time point). Animals on a high fat diet gained more weight than those on standard diet, and wild-type mice gained more weight than ApoE mice (Fig. 1A, $p<0.05$).

Macroscopic differences

There were no obvious differences among groups with regard to the macroscopic appearance of the tissue, with the exception of more peritendinous adipose tissue being observed in C57Bl/6 mice exposed to the high fat diet.

Histology

There were no appreciable differences in the tendon morphology (as assessed on H&E-stained cryosections of Achilles tendon) between mice on normal or high fat diet or between C57Bl/6 and ApoE -/- mice at any time point (0, 15 or 30 weeks). The tenocytes in all experimental groups typically had an elongated shape, with no

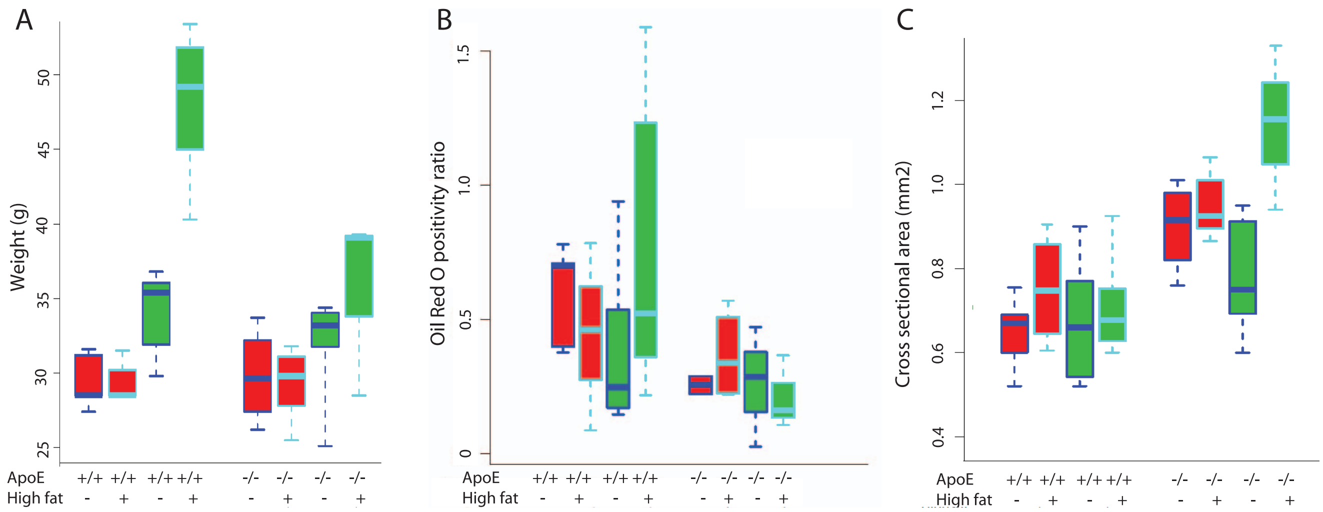

Fig. 1. Effect of diet and mouse type on body weight, tendon lipid content and tendon cross-sectional area. Red bars represent 15 weeks on diet, green 30 weeks. C57Bl/6 and ApoE -/- mice are denoted as "+/+" and "-/-," respectively. A) Mice were weighed at the start and end of the experiment. The weights of all experimental groups were equivalent at the start of the study (not shown). After 15 or 30 weeks, the mice on high fat diet were heavier than those on normal chow, and C57Bl6 mice on a high fat diet gained more weight than ApoE -/- mice ($p > 0.05$). B) Tendon lipid content was quantified as positivity ratio of Oil Red O staining in tendon. Lipid content was significantly different between mouse types, with increased tendon lipid content in C57Bl/6 mice ($p < 0.01$). C). Graph shows the cross-sectional area, measured with grey scale ultrasonography, from the right patellar tendons. Cross-sectional area was significantly different between mouse types, with increased thickness in ApoE -/- mice ($p < 0.05$).

doi:10.1371/journal.pone.0114214.g001

obvious difference in quantity or density. A mild inflammatory cell infiltrate was detected in three tendons from the ApoE -/- strain-KO mice.

Lipid

Oil Red O staining was typically concentrated in and around the tendon tissue (intra- and peri-tendinously), whereas muscle tissue was essentially devoid of appreciable staining. Oil Red O intensely stained adipocytes, particularly in the peritendinous regions of C56Bl/6 mice, but staining was also occasionally observed (in all groups) within both tenocytes and in the collagenous extracellular matrix. Diet (regular vs. high fat) and time (15 vs. 30 weeks) had no effect on the Oil Red O positivity ratio of Achilles tendons. However, the positivity ratio indicated significantly *less* Oil Red O staining in ApoE -/- than C57Bl/6 mice (Fig. 1B). Although unexpected, this finding is in keeping with the fact that ApoE -/- mice gained less weight during the course of the experiment than C57Bl/6 mice (Fig. 1A); this difference in weight gain compared to the C57Bl/6 mice appears to have resulted in less fat being deposited in the peritendinous loose connective tissues and correspondingly a lower Oil Red O tendon positivity score in ApoE -/- mice.

Tendon cross-sectional area (CSA)

Despite the fact that, as reported below, the patellar tendons of ApoE -/- mice were biomechanically weaker and exhibited less peritendinous lipid than patellar tendons of the wild type mice, they had a significantly larger CSA (Fig. 1C). The average CSA of patellar tendons of ApoE -/- mice was 0.97 ± 0.03 mm^2, compared to 0.82 ± 0.03 mm^2 in C57Bl/6 mice ($p < 0.01$). However, diet did not significantly influence the tendon CSA.

Tendon biomechanical function

As an objective indicator of tendon health, we determined the influence of high fat diet on tendon biomechanical properties. Most of the patellar tendons failed at the tibial insertion (91/120), with some failing at the patellar insertion (15/120) and the remainder in the mid-substance (14/120). There was no relation between failure mode and experimental grouping.

The patellar tendons of mice that consumed a high fat diet failed at significantly lower loads (7.92 ± 1.8 N vs 7.0 ± 2.0 N) and stresses (9.31 ± 2.9 MPa vs 10.8 ± 3.9 MPa), compared to those fed a regular chow diet (Fig. 2A–B). In addition, the ApoE -/- patellar tendons failed at a significantly lower stress (9.09 ± 3.9 MPa) than the tendons from wild type mice (11.12 ± 3.0 MPa) (Fig. 2B). There was a statistically significant interaction between diet and mouse type (Fig. 2C). There were no differences among groups for modulus (Fig. 2D), except that older mice (i.e. 30 week vs 15 week time point) displayed a significantly increased modulus (as well as significantly increased stiffness, failure load, and stress).

Finally, there were no differences in the failure strain when comparing mouse types, diets or time points (ApoE -/-: $16.52 \pm 5.94\%$; C57Bl/6: $17.36 \pm 12.0\%$), or in the modulus when comparing mouse types and diet (ApoE -/-: 66.74 ± 31.21 MPa; C57Bl/6: 72.03 ± 35.85 MPa). There were no significant differences in failure load or stiffness between ApoE -/- (6.80 ± 2.35 N, 13.97 ± 5.62 N/mm, respectively) and C57Bl/6 mice (6.81 ± 2.20 N, 12.72 ± 5.46 N/mm, respectively).

Analysis of *Mmp2, Col1a1, Col3a1* and *Tgfb* expression

Mmp2, *Col1a1*, *Col3a1* and *Tgfb* were expressed in the tail tendons of both wild type and ApoE -/- mice (Fig. 3A–C). *Mmp2* expression was significantly increased in mice which had consumed a high fat diet (0.038 ± 0.0061) compared to normal diet (0.064 ± 0.0052) regardless of mouse type. *Col1a1* expression was significantly decreased (5 times lower in log2 ratio) in ApoE -/- mice (0.48 ± 0.13) compared to C57Bl/6 mice (1.08 ± 0.16). There was no significant change in the other genes of interest.

oxLDL in tendons

Immunohistochemistry was conducted using an antibody that reacts with oxLDL (Fig. 3D–F), and in control staining the antibody demonstrated specific

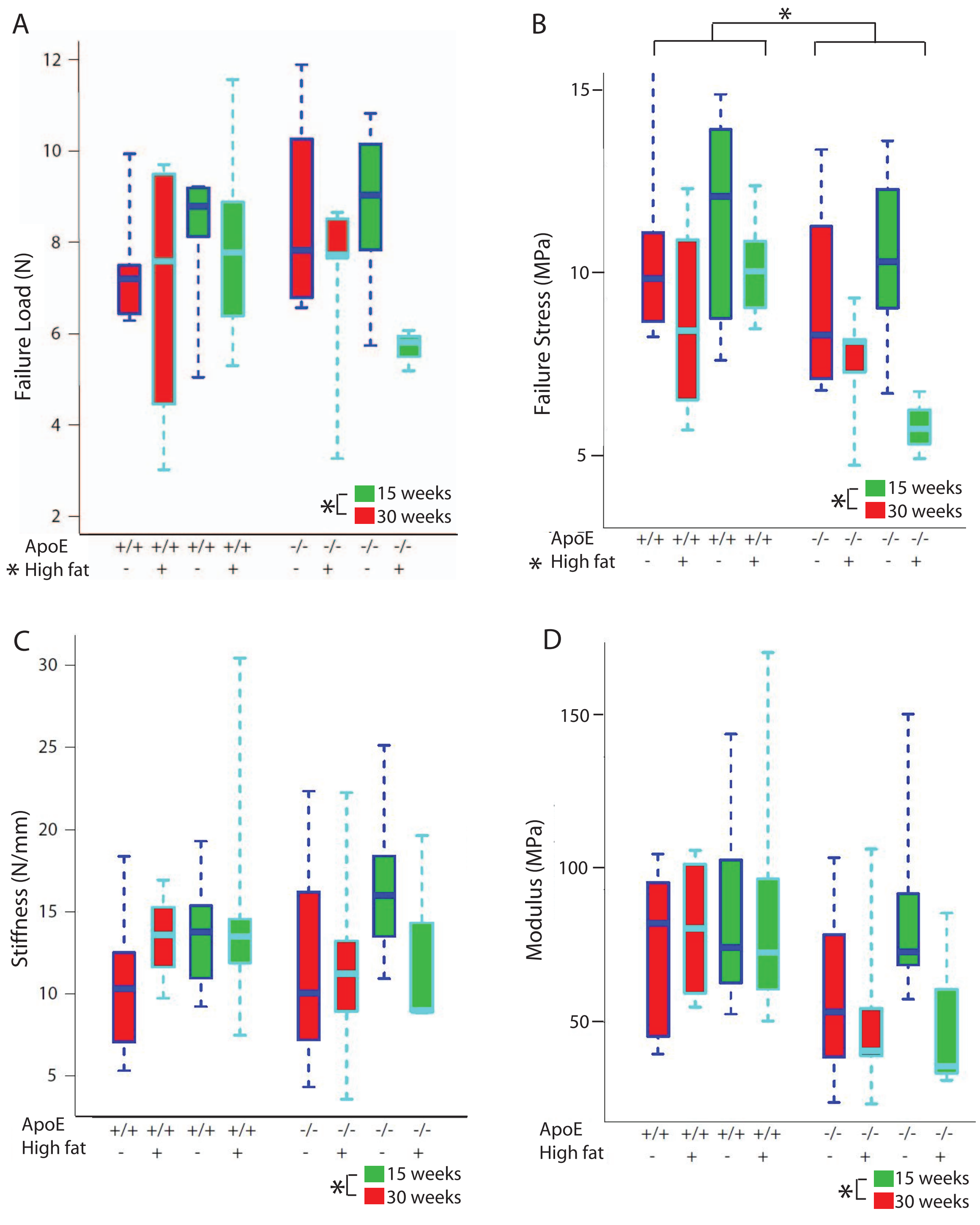

Fig. 2. Effects of diet, mouse type, and time on the biomechanical properties of patellar tendons. Red boxes represent data after 15 weeks on diet, green represent 30 weeks. C57Bl/6 and ApoE -/- mice are denoted as ''+/+'' and ''-/-,'' respectively. Regular chow and high fat diet are symbolized as ''-'' and ''+''. A) Failure load: failure load was significantly lower in the high fat fed mice (denoted with an asterisk, $p<0.05$), but significantly higher in older mice ($p<0.05$). B) Failure stress: failure stress was significantly lower in ApoE -/- mice and those fed high fat diet (denoted with an asterisk, $p<0.05$), and significantly higher in older mice ($p<0.05$). C) Stiffness: stiffness was significantly higher in older mice ($p<0.05$). Within the ApoE -/- mice, those on high fat diet had significantly lower stiffness compared to those on regular chow diet (interaction between mouse type and diet, $p<0.05$). D) Modulus: older mice (30 week time point) had a significantly higher modulus than mice at the 15 week time point ($p<0.05$).

doi:10.1371/journal.pone.0114214.g002

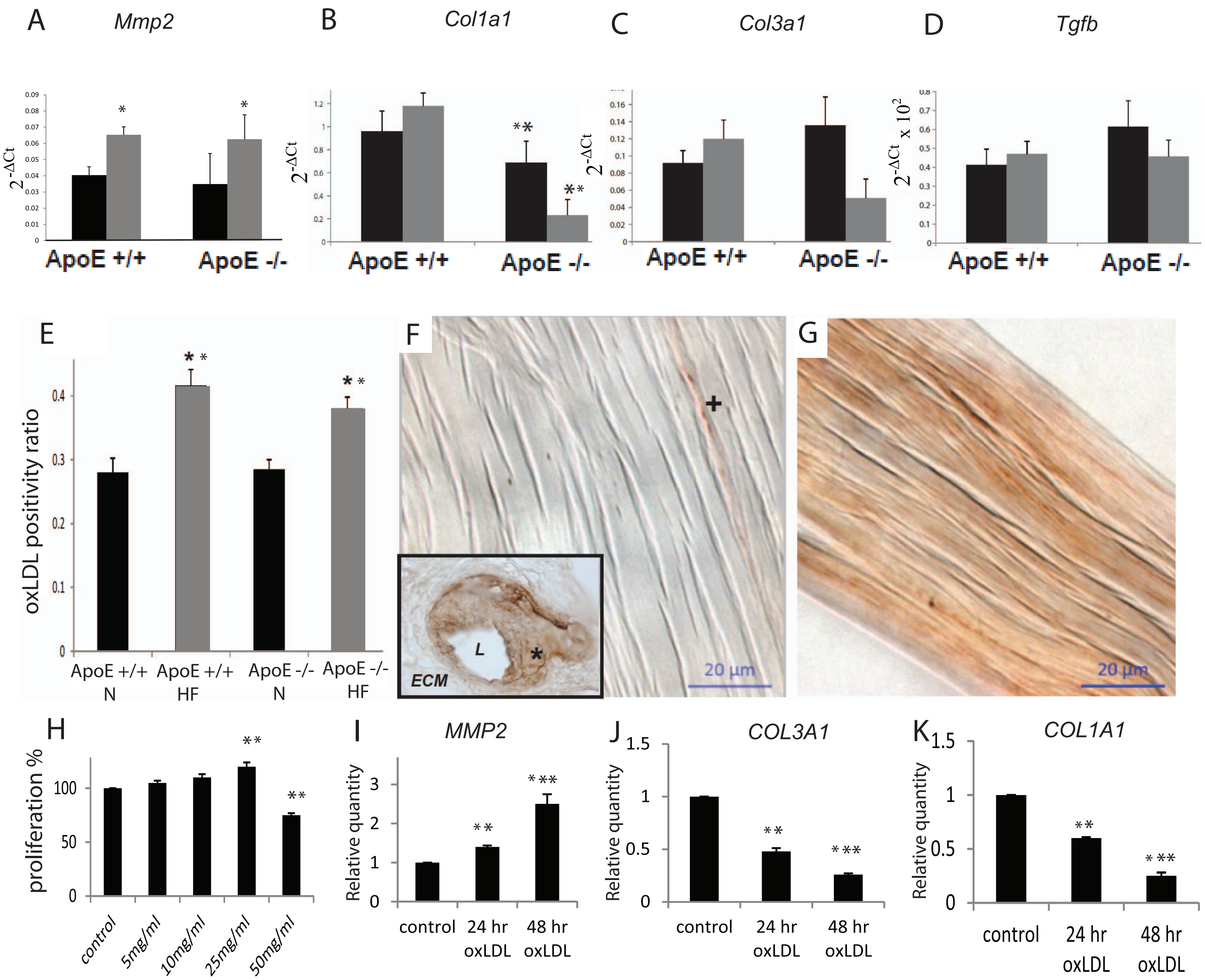

Fig. 3. Effect of diet and mouse type on gene expression (tail tendons) after 30 weeks on normal (black bars) or high fat (grey bars) diet. A–D) *Mmp2* expression was significantly upregulated by consumption of high fat diet, and *Col1a1* expression was significantly lower in ApoE -/- mice, compared to C57Bl/6 mice. E) Effect of diet (30 weeks high fat or normal chow) and mouse type on oxLDL accumulation in Achilles tendon. Tendon OxLDL was higher in mice (ApoE and C57Bl/6) which consumed a high fat diet. F) Note the minimal brown immunolabeling in the Achilles tendon of a mouse on normal diet. The cross demonstrates a small area of staining oriented longitudinally along a collagen fibre. Inset: specific staining in the walls of an atherosclerotic mouse blood vessel. L: lumen; asterisk: positively stained vessel wall; ECM: surrounding unstained extracellular matrix. G) Note the generalized oxLDL immunoreaction throughout the collagenous extracellular matrix of the Achilles tendon from a mouse which consumed high fat diet for 30 weeks. In human tenocytes, (H) 25 ng/ml of oxLDL significantly increased human tenocyte proliferation and (I,J,K) increased *MMP2* expression while simultaneously depressing *COL1A1* and *COL3A1* and after 24 or 48 hours. *$P<0.05$ ** $P<0.01$ *** $P<0.001$.

doi:10.1371/journal.pone.0114214.g003

localization in the tunica intima and media of atherosclerotic blood vessels. In tendons, the immunoreaction was present as a gradation of staining in the load-bearing collagen regions of the tendon proper. Quantification of the oxLDL immunoreaction resulted in a surprisingly clear-cut, and statistically significant, effect of high fat diet: oxLDL accumulated in the tendon extracellular matrix specifically in animals consuming a high fat diet, regardless of the type of mouse.

oxLDL effect on human tenocytes

Given that oxLDL was observed to accumulate in the tendons of mice that consumed a high fat diet, we next examined its influence on human tenocyte (tendon fibroblast) function. OxLDL demonstrated an unexpected ability to slightly but significantly increase the proliferation of human tendon fibroblasts at 25 ng/ml, although at the highest dose tested (50 ng/ml) it was found to be toxic (Fig. 3G). It also induced the differential regulation of genes related to maintenance of the tendon extracellular matrix; *COL1A1* and *COL3A1* (encoding for proteins which are major components of the load-bearing collagen fibrillar matrix of tendon) were decreased in oxLDL-treated tenocytes relative to control cultures, whereas *MMP2* (a gene encoding for an enzyme with collagenolytic activity) was increased (Fig. 3H–J).

Discussion

In this study, we found that oxLDL accumulated in the tendons of mice consuming a high fat diet, and this accumulation was accompanied by an increased expression of *Mmp2* and a reduction of structural and material properties (failure load and failure stress).

Human tendon cells exposed to oxLDL demonstrated a shift in phenotype, reducing their expression of collagen genes (for type I and III fibrillar collagen) and increasing their expression of *MMP2*. We conclude that consumption of a high fat diet can impair the structure and function of tendon tissue and cellularity, and might predispose tenocytes to develop a pathological phenotype through excessive exposure to oxLDL. These observations could shed light on previously reported associations between hypercholesterolemia or increased LDL and tendon rupture [19], or tendinopathy [4], but further research is required to definitively identify a clinically validated risk factor (e.g. fatty diet vs increased LDL) for specific tendon injuries or conditions. *MMP2* has also been shown to be upregulated in an animal model of tendinopathy [13] and in tendinopathy patients [20].

C57Bl/6 mice which consumed a high fat diet gained more weight than ApoE -/- mice, and also demonstrated more peri-tendinous lipid deposition. We speculate that it may be physiological for fat to be stored in the loose connective tissue around tendons, an area sometimes referred to as *mesotendon*. Adipose tissue was indeed observed macroscopically, especially from the tendons of C57Bl/6 mice that consumed a high fat diet for 30 weeks.

Although peritendinous lipid deposition was not as extensive in ApoE -/- mice, they nevertheless exhibited an increased tendon CSA and reduced *Col1a1*. The mechanisms underlying these changes in ApoE-/- tendons are not known at the present time. One possibility is that they result from the known susceptibility to inflammation of this mouse type [21]. In line with this reasoning, PGE_2 administration to tencoytes reduces collagen expression and increases MMP expression and activity – similar effects to those observed with exposure of

tenocytes to oxLDL in this study [18]. To further explore this possibility, future studies could attempt to use more sensitive methods to detect inflammatory substances in the tendons of ApoE -/- mice, as we only detected scant histological evidence of inflammatory cells in the Achilles tendons of these animals. Following another line of reasoning, it has been previously observed that lipid droplets accumulate through direct interaction with the extracellular matrix in the aorta of ApoE -/- mice [22]. This association of oxLDL with the extracellular matrix occurred even in the absence of (i.e. prior to the appearance of) inflammatory cells, and was therefore speculated to represent a primary mechanism by which atherosclerosis is initiated. Given the relative dearth of macrophages observed in the tendons of ApoE -/- mice, the existence of a mechanism by which lipid accumulates directly in the tendon extracellular matrix may be even more revealing than an investigation of inflammatory pathways.

Biomechanical testing of patellar tendons demonstrated a significant effect of high fat diet on tendon function (failure load and failure stress). Previous literature on the biomechanical influences of diet and/or hypercholesterolemia is limited. Zhou et al examined the biomechanics of ApoE -/- mice receiving a Western Diet, with or without one of four nutritional supplements; no differences were found in biomechanical properties among groups, however the data were not shown [23]. Another study reported an *increase* in the tendon stiffness and modulus in the supraspinatus tendons of hypercholesterolemic (ApoE -/-) mice compared to control mice [24], whereas a subsequent study by the same group reported a *reduced* modulus in the patellar tendons of aging ApoE -/- mice compared to controls [25]. Boivin et al studied the influence of high fat diet on C57Bl/6 female mice, and reported a reduced modulus and increased CSA of the Achilles tendon compared to standard diet [26]. It is possible that the increased CSA observed in the study by Boivin et al (measured at the surface of the tendon with digital calipers) resulted from the deposition of peritendinous fat, which was excluded from our US-based CSA measures. Despite the differences in methods between our study and Boivin's, both are consistent in that high fat led to a loss of tendon biomechanical function.

This study has several limitations. First, we were not able to monitor mouse activity levels, and it is possible that activity levels may have influenced tendon properties. In humans, although the consumption of a high fat diet often coincides with a sedentary lifestyle, this is not always the case: a notable exception being athletes who compensate for high levels of caloric expenditure with a high-fat diet. Ideally, future studies will isolate these two variables (diet and physical activity) to determine their relative contribution to tendon properties and/or pathophysiology. Second, our *in vitro* experiments do not allow us to identify the specific molecular pathway by which oxLDL increased MMP2 and COl1A1 expression. Native LDL is associated with a number of proteins which may have contributed to the observed effects. Finally, our experimental design required us to use distinct tendons for different assays; it would be preferable in future, more extensive, studies to conduct all of these assays on a variety of clinically relevant tendons (both upper and lower extremity). More extensive studies of various

tendons is particularly important given the prevalence and morbidity associated with conditions such as rotator cuff tendinopathy, lateral elbow tendinopathy, and Achilles tendinopathy, conditions which involve anatomically and functionally distinct tendons.

Conclusions

The consumption of a high fat diet led to significant, pathophysiological tendon alterations, including increased *Mmp2* expression, accumulation of oxLDL in the extracellular matrix, and reduced biomechanical properties. OxLDL caused tenocytes to adopt a proliferative, degradative phenotype with reduced *COL1A1* and *COL3A1* and increased *MMP2* expression. Further work is required to determine if oxLDL accumulation in tendons may be reversible with appropriate therapy or lifestyle modifications such as diet or exercise.

Supporting Information

S1 Checklist. ARRIVE checklist.
doi:10.1371/journal.pone.0114214.s001 (PDF)

Author Contributions

Conceived and designed the experiments: NG GMT HB A. Sharma AL PZ DJG A. Scott. Performed the experiments: NG HB A. Sharma AL PZ. Analyzed the data: NG GMT HB A. Sharma AL PZ A. Scott. Contributed reagents/materials/analysis tools: PZ WDR DJG. Wrote the paper: NG GMT HB A. Sharma A. Scott AL PZ WDR DJG.

References

1. **Gaida JE, Ashe MC, Bass SL, Cook JL** (2009) Is adiposity an under-recognized risk factor for tendinopathy? A systematic review. Arthritis Rheum 61: 840–9.
2. **Teslovich TM, Musunuru M, Smith AV, Edmondson AC, Stylianou IM, et al.** (2010) Biological, clinical and population relevance of 95 loci for blood lipids. Nature 466: 707–13.
3. **Ozgurtas T, Yildiz C, Serdar M, Atesalp S, Kutluay T** (2003) Is high concentration of serum lipids a risk factor for Achilles tendon rupture? Clin Chim Acta 331: 25–8.
4. **Abboud JA, Kim JS** (2010) The effect of hypercholesterolemia on rotator cuff disease. Clinical orthopaedics and related research 468: 1493–7.
5. **Hirata Y, Okawa K, Ikeda M, Seike M, Matsumoto M, et al.** (2002) Low density lipoprotein oxidized in xanthoma tissue induces the formation and infiltration of foam cells. Journal of dermatological science 30: 248–55.
6. **van Ree JH, Gijbels MJ, van den Broek WJ, Hofker MH, Havekes LM** (1995) Atypical xanthomatosis in apolipoprotein E-deficient mice after cholesterol feeding. Atherosclerosis 112: 237–43.
7. **Riley GP, Harrall RL, Constant CR, Chard MD, Cawston TE, et al.** (1994) Glycosaminoglycans of human rotator cuff tendons: changes with age and in chronic rotator cuff tendinitis. Ann Rheum Dis 53: 367–76.

8. **Sommerville LJ, Kelemen SE, Ellison SP, England RN, Autieri MV** (2012) Increased atherosclerosis and vascular smooth muscle cell activation in AIF-1 transgenic mice fed a high-fat diet. Atherosclerosis 220: 45–52.

9. **Bachem MG, Wendelin D, Schneiderhan W, Haug C, Zorn U, et al.** (1999) Depending on their concentration oxidized low density lipoproteins stimulate extracellular matrix synthesis or induce apoptosis in human coronary artery smooth muscle cells. Clinical chemistry and laboratory medicine: CCLM/FESCC 37: 319–26.

10. **Lusis AJ, Fogelman AM, Fonarow GC** (2004) Genetic basis of atherosclerosis: part I: new genes and pathways. Circulation 110: 1868–73.

11. **Chen KC, Wang YS, Hu CY, Chang WC, Liao YC, et al.** (2011) OxLDL up-regulates microRNA-29b, leading to epigenetic modifications of MMP-2/MMP-9 genes: a novel mechanism for cardiovascular diseases. FASEB journal: official publication of the Federation of American Societies for Experimental Biology 25: 1718–28.

12. **Pasternak B, Schepull T, Eliasson P, Aspenberg P** (2010) Elevation of systemic matrix metalloproteinases 2 and 7 and tissue inhibitor of metalloproteinase 2 in patients with a history of Achilles tendon rupture: pilot study. British journal of sports medicine 44: 669–72.

13. **Attia M., Huet E, Gossard C, Menashi S, Tassoni MC, et al.** (2013) Early events of overused supraspinatus tendons involve matrix metalloproteinases and EMMPRIN/CD147 in the absence of inflammation. The American journal of sports medicine 41: 908–17.

14. **Otsu N.** (1979) A Threshold Selection Method from Gray-Level Histograms. IEEE Transactions on Systems, Man and Cybernetics. SMC-9: 62–66.

15. **Sharma A, Abraham T, Sampaio A, Cowan M, Underhill TM, et al.** (2011) Sodium cromolyn reduces expression of CTGF, ADAMTS1, and TIMP3 and modulates post-injury patellar tendon morphology. J Orthop Res 29: 678–83.

16. **Sereysky JB, Andarawis-Puri N, Jepsen KJ, Flatow EL** (2012) Structural and mechanical effects of in vivo fatigue damage induction on murine tendon. Journal of orthopaedic research: official publication of the Orthopaedic Research Society 30: 965–72.

17. **Havel RJ, Eder HA, Bragdon JH** (1955) The distribution and chemical composition of ultracentrifugally separated lipoproteins in human serum. The Journal of clinical investigation 34: 1345–53.

18. **Behzad HA, Sharma A, Mousavizadeh R, Lu A, Scott A** (2013) Mast cells exert pro-inflammatory effects of relevance to the pathophyisology of tendinopathy. Arthritis research & therapy 15: R184.

19. **Mathiak G, Wening JV, Mathiak M, Neville LF, Jungbluth K** (1999) Serum cholesterol is elevated in patients with Achilles tendon ruptures. Archives of orthopaedic and trauma surgery 119: 280–4.

20. **Alfredson H, Lorentzon M, Backman S, Backman A, Lerner UH** (2003) cDNA-arrays and real-time quantitative PCR techniques in the investigation of chronic Achilles tendinosis. J Orthop Res 21: 970–5.

21. **Huebbe P, Lodge JK, Rimbach G** (2010) Implications of apolipoprotein E genotype on inflammation and vitamin E status. Molecular nutrition & food research 54: 623–30.

22. **Tamminen M, Mottino G, Qiao JH, Breslow JL, Frank JS** (1999) Ultrastructure of early lipid accumulation in ApoE-deficient mice. Arteriosclerosis, thrombosis, and vascular biology 19: 847–53.

23. **Zhou J, Moller J, Danielsen CC, Bentzon J, Ravn HB, et al.** (2001) Dietary supplementation with methionine and homocysteine promotes early atherosclerosis but not plaque rupture in ApoE-deficient mice. Arteriosclerosis, thrombosis, and vascular biology 21: 1470–6.

24. **Beason DP, Hsu JE, Marshall SM, McDaniel AL, Temel RE, et al.** (2013) Hypercholesterolemia increases supraspinatus tendon stiffness and elastic modulus across multiple species. Journal of shoulder and elbow surgery/American Shoulder and Elbow Surgeons… [et al.] 22: 681–6.

25. **Beason DP, Abboud JA, Kuntz AF, Bassora R, Soslowsky LJ** (2011) Cumulative effects of hypercholesterolemia on tendon biomechanics in a mouse model. Journal of orthopaedic research: official publication of the Orthopaedic Research Society 29: 380–3.

26. **Boivin GP, Platt KM, Corbett J, Reeves J, Hardy AL, et al.** (2013) The effects of high-fat diet, branched-chainamino acids and exercise on female C57BL/6 mouse Achilles tendon biomechanical properties. Bone & joint research 2: 186–92.
